# Mental Health Information Reporting Assistant (MHIRA)—an open-source software facilitating evidence-based assessment for clinical services

**DOI:** 10.1186/s12888-023-05201-0

**Published:** 2023-10-02

**Authors:** Ronan Zimmermann, Jon Konjufca, Peter Sakejo, Mrema Kilonzo, Yamil Quevedo, Kathrin Blum, Edison Biba, Tumaini Mosha, Marianne Cottin, Cristóbal Hernández, Sylvia Kaaya, Aliriza Arenliu, Alex Behn

**Affiliations:** 1 Psychiatric University Hospitals of Basel, Basel, Switzerland; 2 Faculty of Psychology, University of Basel, Basel, Switzerland; 3 University of Prishtina “Hasan Prishtina”, Pristina, Kosovo; 4 Muhimbili University of Health and Allied Sciences, Dar Es Salaam, Tanzania; 5 Millennium Institute for Depression and Personality Research (MIDAP), Santiago, Chile; 6 Institute for Implementation Science in Health Care, University of Zurich, Zurich, Switzerland; 7 Leeto Digital Agency, Tirana, Albania; 8 Codeblock, Dar Es Salaam, Tanzania; 9 Universidad Finis Terrae, Santiago, Chile; 10 Universidad Adolfo Ibáñez, Santiago, Chile; 11 Pontificia Universidad Católica de Chile, Santiago, Chile

**Keywords:** Evidence-based assessment, Open-source, Mental health, Software, Digital, Implementation sciences, Electronic health record, Low- and middle-income countries

## Abstract

Evidence-based assessment (EBA) in mental health is a critical aspect of improving patient outcomes and addressing the gaps in mental health care. EBA involves the use of psychometric instruments to gather data that can inform clinical decision-making, inform policymakers, and serve as a basis for research and quality management. Despite its potential, EBA is often hindered by barriers such as workload and cost, leading to its underutilization. Regarding low- and middle-income countries (LMIC), the implementation of EBA is recognized as a key strategy to address and close the prevalent mental health treatment gap.

To simplify the application of EBA including in LMIC, an international team of researchers and practitioners from Tanzania, Kosovo, Chile, and Switzerland developed the Mental Health Information Reporting Assistant (MHIRA). MHIRA is an open-source electronic health record that streamlines EBA by digitising psychometric instruments and organising patient data in a user-friendly manner. It provides immediate and convenient reports to inform clinical decision-making.

The current article provides a comprehensive overview of the features and technical details of MHIRA, as well as insights from four implementation scenarios. The experience gained during the implementations as well as the user-feedback suggests that MHIRA has the potential to be successfully implemented in a variety of clinical contexts and simplify the use of EBA. However, further research is necessary to establish its potential to sustainably transform healthcare services and impact patient outcomes.

In conclusion, MHIRA represents an important step in promoting the widespread adoption of EBA in mental health. It offers a promising solution to the barriers that have limited the use of EBA in the past and holds the potential to improve patient outcomes and support the ongoing efforts to address gaps in mental health care.

**Supplementary Information:**

The online version contains supplementary material available at 10.1186/s12888-023-05201-0.

## Background

The current article is a ‘software article’ [1] presenting the software Mental Health Information Reporting Assistant. The ‘ Background’ section will explain the relevant context and the specific issues that the software described is intended to address. The ‘ Implementation’ section includes a description of the overall features of the software implementation, along with details of any critical issues and how they were addressed. In the results section, the article describes four implementation scenarios in different countries. Finally, the ‘ Conclusion’ section will be followed by a software related ‘Availability and Requirements’ section.

### Importance of evidence-based assessment in mental health care

Mental disorders have a high prevalence and contribute one of the highest global burdens of disease [2]. The mental health treatment gap, defined as those with mental health care needs who are not receiving treatment, is significant in low- and middle-income countries (LMIC) [3]. Cost-effective strategies to address this gap in lower resource contexts are urgently needed [4, 5]. The diagnostic gap, which refers to the proportion of the population with a mental health condition who remain undiagnosed, is an important aspect of the mental health treatment gap [6].

Evidence-based assessment (EBA) for clinical and research purposes relies on unstructured, structured or semi-structured interviews and on questionnaires that determine presence, severity, frequency, and duration of a broad range of psychiatric symptoms. Questionnaires can be filled in by the patient, by mental health care workers or other informants e.g., the family or caregivers of the patient. These instruments are often based on classification systems such as the Diagnostic and Statistical Manual of Mental Disorders (DSM-5) [7] or the International Classification of Diseases (ICD-11) [8]. Alternatively, they can be based on frameworks such as Research Domain Criteria (RDoC) and the Hierarchical Taxonomy of Psychopathology (HiTOP) [9, 10].

Focused on the patient, EBA can act as a structuring principle and tool for decision-making. It supports screening, diagnosis, clinical case formulation, intervention planning, and treatment monitoring [11, 12]. The scientific literature shows that proper use of EBA improves treatment outcomes [13, 14]. In LMIC, EBA can support task-shifted mental health care, i.e., involving non-specialists or lay counsellors to deliver care, under supervision [4]. In addition, EBA can have a positive impact on multiple stakeholder levels including the facilitation of administrative processes, health care provider quality management, informing of regional and national policy makers, and epidemiological research [15]. As such, facilitating and disseminating EBA including in LMIC is one of the cornerstones to address the global mental health treatment gap [6].

### Barriers in uptake and usage of evidence-based assessments

Despite its clearly demonstrated potential and while it is seen as favourable by clinicians, EBA remains largely underused in mental health services both in LMIC and high-income countries [16, 17]. This underusage of EBA is in clear contrast with EBA as a best practice approach [11, 12] and opens the question of the barriers that prevent its usage and implementation. Lewis et al. [15] provide a narrative review on implementing measurement-based care and show how the barriers occur at multiple levels including the patient, practitioner organisation, and the system levels. Among these barriers are lack of knowledge about available and appropriate tools, lack of EBA training, logistic challenges in the procurement of EBA, costs associated with its practice (licences), and clinicians’ attitudes and beliefs regarding EBA. Foremostly the lack of time and human resources during clinical practice to collect data, calculate scales and subscale scores, looking up normative values and interpreting the results is a major barrier. Many of the inefficiencies of EBA are related to its usually paper-based format. While data collection on paper can be convenient and familiar, the subsequent storage and retrieval as well as processing of the data is cumbersome. In our project, we hypothesise that many of the barriers of EBA can be overcome with well-designed digital solutions. To facilitate the use of EBA by shifting to digital systems, we initiated the MHIRA project.

### Aims of the Mental Health Information Reporting Assistant (MHIRA)

Digitalization is a foundation for data-driven improvements of health care services, for example, through data-driven research and improved quality management. However, electronic tools are currently not typically used for mental health clinical work at the scale that their potential would suggest [18].

Electronic health records (EHRs) are digital versions of patients' paper charts, providing real-time, patient-centred information securely to authorised users [19]. They usually contain medical and treatment histories. EHRs can allow access to evidence-based tools that providers can use to make decisions about patients' care. By definition, EHRs also automate and streamline provider workflows. Benefits of using EHRs include improved quality of care, timely access to patient information, and the facilitation of knowledge exchange in multidisciplinary teams. They can also facilitate adherence to clinical guidelines. There are a number of open-source EHR which are popular in LMIC [20]. However, it is crucial that EHR-related processes are designed to be meaningful for patient care and mental health services have specific requirements [21]. Integration of EBA is of high interest for a mental health EHR as it can promote and streamline the use of EBA as shown in high resource contexts [22].

Our aim was to design and develop an open-source digital solution for mental health which facilitates the usage of evidence-based and data-driven practices including in LMICs. The MHIRA project aims at improving the quality of mental health by primarily addressing the needs of front-line healthcare workers. Different mental healthcare settings have unique contexts defined by the populations they address and the disorders they service. Therefore, the envisioned software solution should be versatile and easily adaptable to accommodate this wide range of mental healthcare environments. The contents of the software should be flexibly adaptable to the context-specific requirements in the sense that users can use the EBA instruments of their choice. In line with the digital development principle “reuse and improve” (https://digitalprinciples.org), the content integrated into MHIRA should be already available and psychometrically validated EBA instruments. Psychometric instruments are often developed with academic funding and many authors agree to a free digital usage of their instruments under the condition that their copyright is correctly attributed [23].

To enable data-driven and shared decision making for patients and healthcare workers, the information collected should be accessible immediately in a useful and simple to read format. For these reasons, the main feature of MHIRA is the capability to automatically provide informative and relevant reports to mental health care workers (the ‘Reporting’ in MHIRA). Regarding reporting, the aim was to leverage the available popular data science tools available in R [24] to customise the reports and to enable more complex analyses in clinical contexts. While these tools require some coding skills, they offer maximum flexibility due to the many available libraries and packages [25–27]. Furthermore, R is part of many university-level life science curricula. Therefore, we primarily focused on R as the software language for outputting reports as an initial approach.

### How was MHIRA developed?

To address usability, feasibility, and integration with workflows, we used human-centred design (HCD) [28]. HCD is an approach to problem-solving and innovation that prioritises the needs, behaviours, and preferences of the people who will ultimately use or be affected by a product, system, or service. It involves empathising with the target users, their goals, and their context, and using that understanding to inform the entire design process. The key principle of human-centred design is to involve users throughout the design and development stages, ensuring their active participation and feedback. HCD seeks to create solutions that are intuitive, usable, and meaningful to the users, ultimately enhancing their experience and satisfaction. Additionally, HCD needs to take technological capabilities and economic feasibility into account. HCD has become important in global health and matters for global health equity [29]. HCD emphasises the participation of stakeholders and supports human skills and human values. It focuses on people's needs in the design of complex systems. To develop MHIRA, mental health care workers, software developers, and clinical researchers from Tanzania, Chile, Kosovo and Albania worked together. Combining these settings and conducting the project on a global scale was opted for with the idea that global scaling enables cross-pollination of design and implementation practices, which can improve the sustainability of tech-enabled health systems [30]. We guided the development process by the principles of digital development (https://digitalprinciples.org). We also reviewed the regulatory requirements of the contexts. At each location, the team involved different stakeholders like IT departments, data regulation officers, hospital management, and health policymakers.

The practical process of designing and developing MHIRA was cyclical and iterative as is typical for HCD. It encompassed the following stages:

- Empathise: Gain a deep understanding of the users' needs, goals, and challenges through research, observation, and interviews.
- Define: Clearly articulate the problem or opportunity based on the insights gained from the empathise stage.
- Ideate: Generate a wide range of possible solutions or design concepts, encouraging creativity and brainstorming.
- Prototype: Create low-fidelity representations of the potential solutions to gather feedback and refine ideas.
- Test: Evaluate the prototypes with users, collect their feedback, and iterate on the designs.
- Implement: Develop the final solution based on the feedback and insights gained from testing.
- Evaluate: Continuously assess and refine the solution after its implementation, taking user feedback into account.

These activities were highly organic in terms of the participating team members (also to compensate for restrictions during the COVID-19 lockdowns) and could take many forms such as weekly online meetings, weekly local meetings, international workshops, interviews with the site coordinators, team reflections via google forms and interactive drawing boards, development of concepts by the user experience designers, presentation of technical possibilities by the engineering team, user testing organised by an external company and executed in the local sites. The software engineering process was conducted relying on the agile framework SCRUM [31]. For the coordination of the international team, centralising the information, effective decision making and prioritisation of features, the role of ‘product owner’ [32] was essential.

The implementation of MHIRA is an ongoing effort that has greatly enriched our HCD approach by providing invaluable real-world user insights. As healthcare workers and other stakeholders continue to use the system in their daily routines, they are consistently identifying key usability issues and workflow challenges that may not have been apparent during the initial design and testing phases. This immediate and continuous user feedback is instrumental in driving iterative improvements to MHIRA, making our development process not just theoretically cyclical but dynamically responsive to actual user needs in real-time. These ongoing efforts have significantly increased the efficiency of our HCD process, underscoring the importance of sustained user engagement for the continued improvement and success of tech-enabled health systems.

Finally, MHIRA aims to be accessible as a global digital good. Therefore, the source code is accessible under an open-source licence (http://www.mhira.app).

## Implementation

In this implementation section, we provide an overview focusing on MHIRA’s features including some technical details. Please refer to MHIRA’s documentation (https://mhira.app) for further details about how to use features with screenshots of the software.

### MHIRA is a web-application

MHIRA is a web-application. This means that the software is not intended to be installed on private computers. Instead, MHIRA is accessed via internet browser (e.g., Edge, Chrome or Firefox) by entering the Uniform Resource Locator (URL) of MHIRA. For example, a fictional URL could be https://mhira.my-hospital.org.

### Installing MHIRA with docker

MHIRA needs to be hosted at a trusted service. This can be either a physical device like a server or a cloud computing service. MHIRA can run in different environments and on different operating systems. It is advised to consult a system administrator for its operation. MHIRA encompasses a frontend and a backend and is based on different open-source software packages (see Supplementary Information 1). This can lead to problems with the interplay of different versions of these packages. To address those issues, each of the components of MHIRA is deployed in a Docker container [33] running the required software at a fixed version. All software packages are provided as official images on the Docker Hub Container Image Library [34]. Similarly, the MHIRA frontend and backend are available as images in this library (https://hub.docker.com/u/mhiraproject) in incremental versions.

To orchestrate the different containers, Docker Compose is used [35]. The Docker Compose setup to install MHIRA can be found in this repository: https://github.com/mhira-project/mhira-docker. A file with environment variables allows for customising MHIRA to the requirements of the service (e.g., setting the domain at which MHIRA will be served or the email client it is connected to). The installation guide can be found here and will provide more details: https://bit.ly/3NbgqmA.

With the docker setup, MHIRA can be installed on most operating systems and devices that are able to run Docker containers and for which Docker Compose is available (e.g., MacOS, Windows, Ubuntu).

### Using MHIRA—basic clinical workflow

**Fig. 1:**
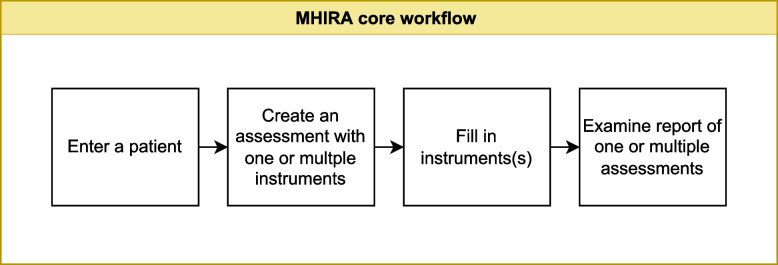
Core workflow

Once MHIRA is installed, the basic clinical workflow in MHIRA consists of 4 steps (Fig. 1). First, a patient profile is created in the system to store all patient-related information. In a second step, assessments, which contain one or multiple digital instruments, are scheduled. Next, the instruments in the assessment are answered by an informant. Finally, a clinician accesses the report which is automatically generated from the collected data.

To illustrate the possible outcomes of the procedure, we have included example reports in Supplementary Information 2, which features a single assessment, and in Supplementary Information 3, which includes a follow-up assessment.

### Patient management

MHIRA is conceptualised as an electronic health record centred around cases (i.e., patients). An Electronic Health Record (EHR) is essentially a digital equivalent of a patient's physical medical chart. EHRs provide real-time, patient-focused data, ensuring instant and secure access to pertinent information for approved individuals [19]. By creating a patient entry, the mental health care workers can access all the information about their patient in one location. The patient entry in MHIRA contains a profile with identifying information about the patient as well as contact information. Case managers can be optionally assigned to the patient, clarifying responsibilities within the clinical team. The possibility to add caregivers was considered important by mental health care workers in our project, especially since the project was mainly targeted at child and youth mental health services. The HCD approach revealed that patients in LMICs are usually accompanied by family members when visiting the services. Therefore, a feature to document caregivers was added to MHIRA. The section about patient management in MHIRA’s user manual provides more details and screenshots: https://bit.ly/3CLW4LP.

### Adding instruments to MHIRA

The clinicians from various services involved in the project have formulated a key requirement for each service: the flexibility to add instruments that best cater to their specific needs. Moreover, the services must adhere to using instruments that have been appropriately translated and validated for their respective cultural contexts. To address these needs, MHIRA was created as a versatile platform that does not come pre-loaded with any specific questionnaires. Instead, it allows users to upload the necessary instruments. To utilize MHIRA effectively for implementing EBA, a mental health care service must first upload a relevant set of instruments, such as psychometric questionnaires, onto the platform. These uploaded instruments will then serve as a resource to aid in the EBA process.

Instruments can be uploaded to MHIRA using the ‘xlsform’ format (https://xlsform.org). Xlsform is a standard for simply authoring forms and used in a number of open-source applications (e.g., Open Data Kit and KoboToolbox). The standard works by defining the instrument items in an Excel file which is then rendered as a form by the software. Getting started with xlsform is simple thanks to the excellent documentation on the above-mentioned website. As a limitation, MHIRA in its current version focuses on the most useful item types of this format i.e., multiple choice with one or multiple answers, text, numbers, and notes.

With this instrument upload system, most validated psychometric instruments can be integrated to MHIRA. Alternatively, custom made instruments can be uploaded (e.g., a form which is relevant for the clinical processes at the service). MHIRA requires entering copyright information on the instruments as this was requested by instrument copyright holders [23]. We recommend obtaining permission from the copyright holders if permission to use and create derivatives is not clear from the copyright statement.

Finally, MHIRA contains a versioning system for the instruments. In case of modifications to uploaded instruments, the old versions are documented in an archive. Collected data is always associated with a specific version of an instrument, allowing to keep track of what version of an instrument was used for data collection. Please refer to the MHIRA manual for more details on uploading instruments to MHIRA: https://bit.ly/3TibPkD.

### Assessments

Assessments are part of the core workflow (see Fig. 1) which aims at facilitating the use of psychometric instruments in an evidence-based assessment framework. After instruments have been uploaded and a patient has been entered, assessments can be planned for a specific patient. See this section of the user manual for details: https://bit.ly/3DbW2gz.

For each assessment, one or multiple instruments may need to be selected. The assessments can be opened directly on the device of the clinician, opened on another device with a camera using a QR code, or the encrypted deep link can be sent to the informant manually or automatically by email.

Assessments can be selected to be either always open for completion or they can be restricted to a specific time frame. For example, an assessment could be planned to be filled in as a follow-up in which case they would be sent via email after a couple of months. Assessments can also be set to expire at a certain date, after which they are no longer accessible.

Once created, the assessments have different statuses: planned, open for completion, partially completed, expired or cancelled. If ‘open for completion’ or ‘partially completed’, the assessments can be accessed.

The recipient of the link will be forwarded to his/her assessment via the encrypted deep link. First, an overview page with all instruments included in the assessment will be shown. For each instrument, the number of answered questions in relation to the questions that are required to finalise the instrument are shown. Once all required questions of all instruments in the assessment are answered, the assessment can be submitted. However, data is written directly to the database at the moment a question is answered. This ensures that no entered data is ever lost, even if the clinician, patient, or any other parties accidentally fail to finalise the assessment. The submission action closes the link and further changes to the instruments are no longer possible.

### Reporting

Once instrument data has been collected, the mental healthcare worker can proceed to access a report on the collected data. As an example, this could be the calculated scales of a given instrument represented as a graph which also shows the likelihood of the results compared to healthy control subjects (see Supplementary information 2). In case of repeated measures, the graph shows the severity of symptoms over time (Supplementary information 3). A report could also link to psychoeducation materials or suggest treatments or further assessments based on the obtained results.

From a user perspective, obtaining a report is done by pressing a reporting button in the reporting section in a specific patient entry. MHIRA does not contain any specific reports as it is conceptualised as a platform to which instruments and reports can and need to be added. For more details on the reports, please see the section below.

### Reporting mechanism

Due to the flexibility in the selection and use of instruments, the project was confronted with the challenge of flexible and adaptive reporting for any given instrument. MHIRA’s strategy to address this issue is leveraging available data science tools like those available in R (e.g., ggplot [26], tidyverse [27], shiny [25]). As MHIRA is installed in the form of orchestrated Docker containers running different modules of the software, software used for reporting can be added to the system of containers. Official docker images of data science software in different configurations are available at the Docker Hub Container Image Library [34].

**Fig. 2:**
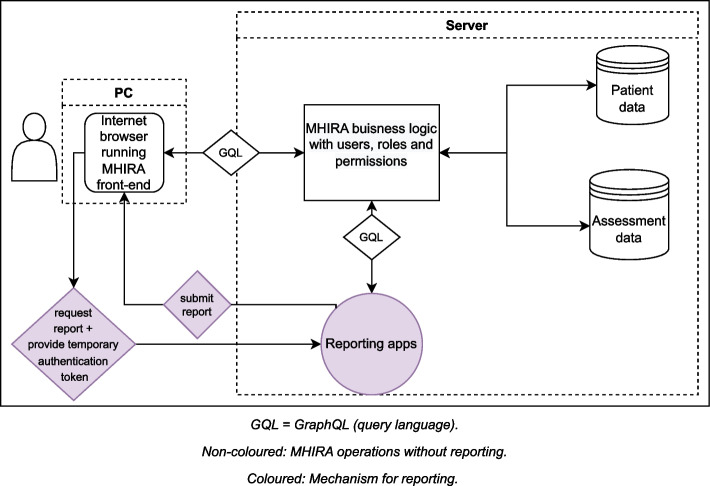
Integration of reporting apps with MHIRA

Figure 2 shows a schematic and simplified overview of how reporting tools interact with the MHIRA business logic. When using MHIRA without a report (e.g., creating a new patient), the user interacts with MHIRA’s front-end. The front-end requests the create, read, update or delete action via GraphQL [36] queries or mutations. The MHIRA business logic in the backend checks the user’s permission to obtain information and interact with the databases. A response is then returned to the user front-end.

Reporting is represented in colour in Fig. 2. A ‘reporting app’ (e.g., a shiny app) receives a request from the front-end to generate a specific report. With this request, the reporting app also obtains a temporal authentication token to retrieve the data required for the report. The reporting app will then request the required data from the MHIRA business logic via GraphQL query language providing the token to authenticate the request. Once the data is obtained, the reporting apps will proceed to build the report and display it in the user’s browser tab. Details about reporting and how to set it up including the management of normative values for the instruments can be found here: https://bit.ly/3stuDBI.

### Permissions and user roles

MHIRA has a number of permissions e.g., viewing patients, managing patients, deleting patients, viewing instruments, managing instruments, viewing assessments, managing assessments, deleting assessments, viewing users, managing users, deleting users, etc. These permissions can be assigned to user roles (e.g., ‘psychiatrist’, ‘nurse’ or ‘user manager’). In turn, one or multiple user roles can be assigned to a given user to grant this user a set of permissions. User roles and their permissions are incremental and have been designed to be suitable to a wide range of clinical and counselling settings. The user roles need to be customised to a given service. More details and examples are provided here https://bit.ly/3gJTiPA (permissions) and here https://bit.ly/3sw4L89 (user roles).

### User management

User accounts are created by a user manager (a user with the permission to view and manage users) defining a username and a password for each user. The credentials need to be communicated by the user manager to the user via a secure channel external to MHIRA. To access MHIRA, users need to login with their username and password. Whenever the password was set by a user manager, the user is prompted to enter a new password which cannot be the same as his/her previous passwords. The user manager can also reset passwords for existing users in MHIRA. By default, a user with access to MHIRA has no user role and, consequently, no permissions except for viewing his/her own profile. The user manager assigns one or multiple user roles to a user.

### Restriction of access to patient information

Each registered patient must be linked to at least one department. Patients and staff members can be assigned to multiple departments. Users can only view patient information if they belong to the same department, ensuring data privacy. The level of access restriction can vary based on the healthcare service's use of this feature. If all patients and users are in the same department, all users can access all patients. Conversely, if each clinician has their own department, only one healthcare worker can access a specific patient. Patients can be ‘transferred’ between departments by adding them to another department. Finally, this system allows for users who do not see any patients in the system, e.g., those who only manage instruments or only manage users.

### Messages and disclaimers

Disclaimers in MHIRA were a requirement by some of the health care providers’ legal departments. As an example, when filling in instruments, the patient and other informants need to understand that it might take a while until the mental health care workers can react to the reported data. This type of message needs to be customised for each service.

## Results

In the following sections, we will present different contexts in which MHIRA was implemented. Table 1 compares these four settings in terms of personnel using MHIRA, the main purpose of using MHIRA, the main data collection mode, number of clients and assessments performed with MHIRA in the pilot phase and the instruments the local staff decided to implement in MHIRA.

**Table 1:** Descriptive information about implementation sites

**Implementation Site**	**San Joaquín Medical Center (CMJS)**	**School Psychology Services in Kosovo**	**Muhimbili National Hospital (MNH)**	**Grupo Mentaliza**
**City, Country**	Santiago, Chile	Pristina, Kosovo	Dar es Salaam, Tanzania	Santiago, Chile
**Type of service**	Outpatient Mental Health Clinic in University Hospital	Elementary Schools with onsite Psychologist	Teaching and Tertiary Referral Hospital	Private Mental Health Clinic
**Personnel using MHIRA**	Psychologists, psychiatrists	School psychologists	Nurses, psychologists, psychiatrists, occupational therapists	Psychotherapists
**Main data collection mode**	Front-desk worker provides assessments on tablets in the waiting room. QR code is scanned with the tablet's camera	At school psychologist's office using tablet or laptop	Assessment is started on a desktop computer in an assessment room or the clinician’s office	Assessment links are sent via email
**Main purpose**	Monitoring of psychotherapy process	Mental health and school aptitude assessments of pupils	Screening & generating electronic patient file	Screening and follow-up
**MHIRA usage in terms of number of clients and assessments in the initial pilot phase**	85 patients with 243 assessments from Nov 2022 to Jan 2023	62 pupils with 82 assessments Sep 2022 to Jan 2023	32 patients with 32 assessments from Aug 2022 to Dec 2022	8 patients with 25 assessments from Nov 2022 to Jan 2023
**Instruments included in the local version of MHIRA**	PHQ-9, GAD-7, CORE-OM, LPFS BF 2.0, PID5BF + BPFSC-11	GDS, AC, HC, DASS21, PHQ-A, PSDQ, BDI, PANAS, MSPSS, Kessler10, SIPDC, IAT, DgC, DlC, DpC, DcD, SCAS-P, SCAS-C, ADDC	K-SADS-PL, PHQ-A, BPFSC-11, LoPF-Q 12—18	PHQ-9, GAD-7, CORE-OM, LPFS BF 2.0, PID5BF + BPFSC-11

*PHQ-9* Patient Health Questionnaire [37], *GAD-7* Generalized Anxiety Disorder Questionnaire [38], *CORE-OM* Clinical Outcomes in Routine Evaluation-Outcome Measure [39], *LPFS BF 2.0* Levels of Personality Functioning Brief Form [40], *PID5BF + * Personality Inventory for DSM-5 Brief Form [41], *K-SADS-PL* Kiddie Schedule for Affective Disorders and Schizophrenia [42], *PHQ-A* Patient Health Questionnaire for Adolescents [43], *BPFSC-11* Borderline Personality Features Scale for Children [44], *GDS* Goldberg Depression Scale [45], *AC* Checklisft for Autism [46], *HC* Hyperactivity checklist [46] *DASS21* Depression, Anxiety, and Stress Scale 21 [47], *PSDQ* The Parenting Styles and Dimensions Questionnaire [48], *BDI* Beck Depression Inventor [49], *PANAS* The positive and negative affect schedule [50], *MSPSS* Multidimensional Scale of Perceived Social Support [51]; Kessler10 [52], *SIPDC* Checklist for Sensory Information Processing Disorder [46], *IAT* Internet Addiction Test [53], *DC* Dysgraphia Checklist [46], *DlC* Dyslexia checklist [46], *DpC* Dyspraxia checklist [46], *DcC* Dyscalculia checklist [46], *SCAS-P* Spence Children’s Anxiety Scale – Parents’ version [54], *SCAS-C* Spence Children’s Anxiety Scale – Children’s versión [54], *ADDC* Attention Deficit Disorder Checklist [54]

### San Joaquin Medical Centre (CMJS)—Santiago, Chile

CMSJ is the largest centre of the private health network owned by the Pontificia Universidad Católica de Chile and the international health network Christus Health (UC-CHRISTUS Health Network; https://www.ucchristus.cl/). Mental health services are provided by the Mental Health Unit (MHU). CMSJ has a large catchment area located in the south of Santiago de Chile. Because CMSJ is a teaching hospital, services are provided by staff clinicians, by psychiatry residents and psychology interns. The MHU includes many different mental health specialty programs, including: Family Therapy, Infant Psychotherapy Program, Child and Adolescent Psychiatry and Adolescence Mental Health Program among others. Between 2017 and 2018, 8′633 adult psychiatry sessions, 3′433 in paediatric and adolescent psychiatry sessions and 22′601 psychotherapy sessions were held at the MHU. The MHU provides specialised services by fully trained psychiatrists or psychiatry residents and by fully trained psychologists or psychology interns. The MHU does not offer inpatient hospitalisation services. However, these services are available at other locations of the network. MHIRA is being piloted at the MHU, initially focusing on one specific program, namely the Adult Psychotherapy Unit (APU). Generally, in the MHU and specifically in the APU, no standard EBA procedures exist, and the clinicians' attitude towards the use of such procedures is mixed, with some clinicians being more receptive than others. In this regard, MHIRA’s implementation in this setting poses typical challenges for innovation and behavioural change at the level of clinicians and at the same time, important opportunities to improve the quality of the care.

MHIRA was implemented on a virtual machine provided by the UC-CHRISTUS Health Network. It is accessible on the intranet of the hospital or via VPN. MHIRA has been used primarily to deliver a broad-spectrum psychological functioning instrument, namely the CORE-OM [39], which is currently used to track treatment outcomes for patients in individual psychotherapy. Before each psychotherapy session, and with the use of a tablet, clinicians have used MHIRA to deliver the CORE-OM in the waiting room of the MHU. For this purpose, the centre requested a feature which allowed the front desk personnel to submit the assessments via QR Code. Between November 2022 and January 17, 243 assessments were completed for 85 patients, including intake assessments as well as treatment-monitoring assessments. Thus, at the MHU, using MHIRA has been proven to be a feasible and acceptable method to track patient progress in routine care, further signalling positive prospects for routine implementation above and beyond the APU, where it is currently being piloted. One of the problems to solve in this setting is the interoperability with the existing health record system which would help avoid double entries of patient information into MHIRA.

### School psychology services—Pristina, Kosovo

Several elementary and high schools in Kosovo have an appointed school psychologist who supports teachers, pupils, and their parents to facilitate learning, growth, and preventative mental healthcare. The school psychologists mainly provide supervision and guidance for teachers, pupils, and their parents. In some cases, families are referred to healthcare services.

A short-term priority of the education system in Kosovo is to expand the network of school psychologists to other schools in Pristina and throughout the country. Kosovo is composed of seven regions, and each municipality within those regions has its own system of elementary and secondary schools. Currently, Kosovo's school psychologists do not have access to electronic information systems. The implementation of MHIRA is seen as a promising project by the school psychologists as it addresses a procedural gap and has the potential to improve efficiency and workload, optimising the psychologist's role and impact.

As of now, MHIRA is hosted at the University of Pristina on a server provided by the project. Schools can access the system over the internet through a secure connection. The software has been successfully implemented in seven public elementary schools in Kosovo’s capital. The software currently offers 19 free-to-use instruments in Albanian that cover a range of topics including learning disabilities, emotional disturbance, social relationships, and internet addiction, among others (see Table 1). Adding these instruments to MHIRA was suggested by the school psychologists during focus groups. The new use of these instruments enables the school psychologists to have a comprehensive understanding of the pupils' well-being and academic performance. The reports were perceived to help in the communication with parents and the teachers. The successful implementation of MHIRA in Kosovo is likely scalable with the support of the alliance of school psychologists and the department of education.

### Muhimbili National Hospital—Dar es Salaam, Tanzania

In Tanzania, MHIRA has been implemented at the Muhimbili National Hospital (MNH), which is a teaching and tertiary referral hospital serving the catchment population of five administrative districts in Dar es Salaam and receives referrals from other regions of the country. MHIRA is currently accessible at the child and adolescent psychiatric clinic under the Department of Psychiatry and Mental Health. This newly established service is available four days a week and focuses on young patients. The most common disorders treated at the clinic include specific psychiatric functional disorders, behavioural problems associated with intellectual disability, developmental disorders (such as ADHD and Autism), psychological distress or psycho-trauma, and substance use-related problems. The services include assessments, school placement and outreach, biological management, psychological interventions, occupational therapies, parent support groups, and liaison work with ear, nose, and throat specialists (for children with cochlear implants) and urology specialists (for children with ambiguous genitalia). The multidisciplinary staff of the service consists of psychologists, psychiatrists, social workers, occupational therapists, nurses, medical doctors (registrars), a medical records clerk and an accountant. The permanent staff is supported by residents in clinical psychology and psychiatry. MNH has a health management information system that records information needed to plan, provide and evaluate clinical processes. However, this system is used by all clinical departments and does not allow for customisation according to specific needs by a particular specialty. The lack of customised EHR prevents clinicians from having a complete paperless patient information system and hence physical files are still used at the clinic. The use of EBA is determined at the health care provider level in addition to formal clinical assessments. The need to improve access to patient records as well as to standardise EBA for various problems and disorders has been expressed by the clinical staff at the clinic. MHIRA has been installed on a server provided by the project which is located in a secured server room at the hospital. MHIRA is only accessible via the local network from within the hospital. The hospital team implemented the Kiddie Schedule for Affective Disorders and Schizophrenia (K-SADS-PL) [42] with the permission of the authors. Additionally, the Patient Health Questionnaire for Adolescents (PHQ-A) [43] was implemented. As part of a research protocol, the Borderline Personality Features Scale for Children [44] was used in electronic format via MHIRA. The software has been well received for its potential to provide systematic screening and automatic reports. Another perceived advantage is that MHIRA also supports research and training of residents: The latter can now discuss standardised results with their supervisors. The clinicians at MNH see functionality for adding clinical notes to MHIRA as highly desirable. However, at the time of writing this article, this feature has yet to be implemented, thus limiting the full adoption of the software by the service.

### Private health centre for adolescents—Santiago, Chile

Grupo Mentaliza (https://grupomentaliza.cl) is a private mental health centre. Its clinical team specialises in the comprehensive treatment of complex cases of substance use disorders and personality disorders in adults and adolescents. The staff is composed of 4 adult psychiatrists, 2 child psychiatrists, 10 psychotherapists and one occupational therapist. The centre provides individual, family and group interventions. The senior staff members are involved in teaching, mental health research and supervision of other professionals. The team is highly motivated to provide evidence-based practice.

The health centre uses software for administrative purposes like the scheduling of sessions, billing and clinical note taking. However, prior to the implementation of MHIRA, EBAs were not systematically applied. MHIRA is hosted on a local cloud provider for this health centre. As many of the psychotherapeutic sessions at the centre are held via video conferences, it was important that the assessments provided by MHIRA could be sent via email. Additionally, advanced planning and bulk setup of assessment was among the requested features. With this functionality provided, MHIRA is being used by the majority of clinicians at the services. In the period from November 2022 and January 2023, 25 assessments have been completed for 8 patients. MHIRA has been well received by clinicians. The centre is planning to scale up the use of EBA with MHIRA.

## Conclusion

MHIRA is a software designed to simplify EBA in mental health care. Early adoption and implementation across various international settings with varying conditions and target populations, ranging from tertiary teaching hospitals in Chile and Tanzania to school settings in Kosovo and a private group practice in Chile have shown that the software is well-received by mental health care workers. The main benefit of MHIRA is the ability to provide high-quality and convenient mental health care data through the use of instruments that can be selected by the health care workers. Thus, the resulting reports offer relevant information for clinical services and provide an efficient and reliable way to retrieve patient files. Additionally, the platform offers flexibility in delivering assessments to patients through various methods such as QR codes, email, telemedicine, or digitalised structured interviews. Integrating data science tools into MHIRA has the potential to greatly enhance reporting. Advanced models, such as bifactor models for personality instruments, can be utilised to calculate instrument scales [62], while machine learning models can also be integrated [63]. Moreover, reporting apps can support clinical processes and quality management.

So far, MHIRA has been used in mental health screening of patients, therapy monitoring, and research applications and has been found to be valuable in the supervision of trainees. The psychometric data provided by the software adds an additional perspective on clinical cases and can serve as a medium for communication between supervisors and trainees. This highlights the potential of MHIRA in task-shifted mental health care [4]. Furthermore, the reports can assist in the communication between members of interdisciplinary teams [64, 65].

MHIRA's current reporting tool (Supplementary Information 2) enables clinical services to incorporate interpretations and recommendations from questionnaire results. Looking forward, a potential improvement to MHIRA could be the automated generation of personalised psychoeducational reports for patients and their families. Psychoeducation has proven to be an effective therapeutic strategy in mental healthcare [55, 56, 66–68]. Healthcare in LMICs often relies on family and community support, making the provision of psychoeducation critical for instructing the best support strategies for patient disorder management [57, 58]. With the utilization of evidence-based algorithms (EBAs) and advanced language models, the content of psychoeducational reports can be tailored to the individual and their socio-cultural context, thereby improving the effectiveness of psychoeducation [59, 60]. However, even with the promise of mental healthcare support systems built on EBA, the importance of the therapeutic alliance and therapist interactions remain fundamental in mental healthcare [61]. Therefore, MHIRA and EBA should be used as a structuring principle within a healthcare service [12], supplementing but not replacing the essential human component of mental healthcare. While the reporting functionality of MHIRA is a major benefit, entering patient information and setting up assessments are additional processes in the clinical workflow. These additional processes can potentially be burdening in view of increasing data demands in health care [69]. Feedback from health care workers involved in the co-design of MHIRA suggests that the software is already considered user-friendly and acceptable for clinical work. But there might still be room for improvement in terms of user-friendliness and efficiency. One proposed solution is to allow users to self-check-in by completing their own profile after accessing the form via QR code in the facilities' waiting room. Such functionality might be added in the future if the use cases can be confirmed.

Another limitation to consider pertains to our HCD approach. While we engaged extensively with mental healthcare workers, software developers, and researchers in the design process, the perspectives of patients and their caregivers were not directly included. However, it's worth noting that we do receive indirect feedback on patient interactions through healthcare workers. The absence of direct patient and caregiver involvement was partly due to ethical complexities tied to engaging a vulnerable population, which would have required additional consent procedures. Furthermore, the immediate patient interaction with MHIRA is through well-tested, standardized questionnaires, lessening the need for their direct involvement in the initial stages of design. As MHIRA evolves, particularly in the realm of reporting features, the inclusion of these perspectives will become increasingly essential for a more rounded user experience.

In its current implementation, MHIRA exhibits a potential barrier concerning the process of adding instruments and setting up reports with normative values, which may require some training. This issue arises due to MHIRA's deliberate approach of not including pre-loaded instruments. The rationale behind this decision was driven by the significant differences observed among the services participating in our initial co-design project. As each service had unique requirements, there was no consensus on using the same standardized instruments across all services. Thus, the emphasis was placed on providing flexibility, enabling each service to add instruments tailored to their specific needs. This approach aligns with the global scope of the MHIRA project. However, we acknowledge that the need for training and individual instrument set-up can be a challenge for users. To address this concern, a potential solution, as proposed in the review by Cottin et al. [23], could be the creation of an online repository of instruments. Such a repository would offer a centralized hub of validated and standardized instruments, accessible to users within MHIRA. This repository could alleviate the burden of setting up instruments and streamline the process of generating normative reports.

A further current limitation of MHIRA is related to the problem of system interoperability, which refers to the ability of different systems, devices, or software to work together and communicate with each other. This issue has been encountered in three out of the four of the presented use cases. For example, at the MNH in Tanzania, patient information needs to be entered into a main hospital management system for accounting and other hospital management purposes, leading to duplicate entries. The issue can be resolved by integrating MHIRA and other systems via the Health Level-7 standard (HL-7). As MHIRA is an open-source software, users have the possibility to make the changes required for a satisfying integration into the context of the service. Over time, such solutions will hopefully become available to be reused by other services. Some health care services will not accept the implementation of MHIRA because they work with closed proprietary systems or because the cost of integration might be too high.

Finally, functionality such as clinical note taking and managing sessions with the patients (calendar functionality) as well as support for administrative tasks are desirable. The team is working towards providing such features in future versions to improve the impact of MHIRA.

The present article highlights the features of MHIRA, an open-source software developed through an HCD approach in collaboration with clinicians and researchers from diverse cultural backgrounds, including LMICs. While the article provides valuable insights into the software's development process, it does not comprehensively address the implementation of MHIRA, which could benefit from a more rigorous scientific examination supported by quantitative and qualitative data. To further elucidate the real-world application of MHIRA, it is essential to conduct a study that systematically investigates its implementation. Importantly, a study design has already been registered on the Open Science Framework (https://osf.io/j42g3), indicating a forthcoming investigation into this aspect of MHIRA's deployment. The study design will include comprehensive assessments of the software's accessibility, usability, and sustainability. Once the MHIRA software reaches a mature stage with efficient implementation strategies in place, it becomes imperative to conduct a thorough evaluation of the cost of implementation and usage. This analysis is essential to determine whether utilising MHIRA can indeed prove to be a cost-effective solution for services in LMIC. While the usage of MHIRA may incur expenses related to hosting devices, qualified technical personnel, and clinical staff training, we anticipate that MHIRA's cost-effectiveness will surpass that of non-open source EHRs.

## Availability and requirements

*Project name*: Mental Health Information Reporting Assistant.

*Project home page*: https://github.com/mhira-project

*Operating system(s)*: Linux, Windows, Mac OS and any operating system able to run Docker. The software was tested on Ubuntu 21.04.

*Programming language*: TypeScript, R, Dockerfile.

*Other requirements*: Docker (www.docker.com) and Docker-compose (docs.docker.com/compose/).

*Licence*: MPL-2.0

*Any restrictions to use by non-academics*: None.

### Supplementary Information

**Additional file 1: Supplementary information 1.** Programming language and software stack for MHIRA.

**Additional file 2: Supplementary Information 2.** Example of MHIRA report.

**Additional file 3: Supplementary Information 3. **Example of MHIRA report with follow-up.

## Data Availability

This article focuses on presenting a software. No data sets were used.
