# Supplementary material for: Mental Health Information Reporting Assistant (MHIRA)—an open-source software facilitating evidence-based assessment for clinical services
Source: BMC Psychiatry. 2023 Oct 2;23:706. doi: 10.1186/s12888-023-05201-0 (PMC10544613; doi:10.1186/s12888-023-05201-0)
Supplement: Supplementary file 1 — Additional file 1: Supplementary information 1. Programming language and software stack for MHIRA. [file 12888_2023_5201_MOESM1_ESM.docx]

## Supplementary information 1 - Programming language and software stack for MHIRA

The main programming language used for MHIRA is ‘typeScript’ [31]. The software stack consists of:

- Front-end: Angular [32]
- Backend: NestJS [33] which is based on Node.js [34]
- Database 1: MongoDB [35] for the data related to instruments and assessments
- Database 2: PostgreSQL [36] for patient profiles and parameters related to MHIRA’s business logic
- Web Server: Caddy 2 [37]
- Cache: Redis [38]
- Query language for Application Programming Interface: GraphQL [39]
- Containerisation and deployment: Docker [40] and Docker-compose [41].
- Version control and repository: Github [42]

A description of the source code would go beyond the scope of the current article. MHIRA is open source: <https://github.com/mhira-project>.
