# Supplementary material for: Mental Health Information Reporting Assistant (MHIRA)—an open-source software facilitating evidence-based assessment for clinical services
Source: BMC Psychiatry. 2023 Oct 2;23:706. doi: 10.1186/s12888-023-05201-0 (PMC10544613; doi:10.1186/s12888-023-05201-0)
Supplement: Supplementary file 2 — Additional file 2: Supplementary Information 2. Example of MHIRA report. [file 12888_2023_5201_MOESM2_ESM.pdf]

Supplementary Information 2 – Example of MHIRA report

MHIRA ≡

medicalRecordNo	initials	age	gender
ML006	ML	25.4	female

PHQ-9

PHQ-9

version: 64bbbbb3a1f9b1002a5a0b67

The PHQ-9 measures depression severity. It is responsive to changes of condition severity over time.

The manual can be found here.

The PHQ-9 was developed by Drs. Robert L. Spitzer, Janet B.W. Williams, Kurt Kroenke and colleagues, with an educational grant from Pfizer Inc. No permission required to reproduce, translate, display or distribute.

Figure

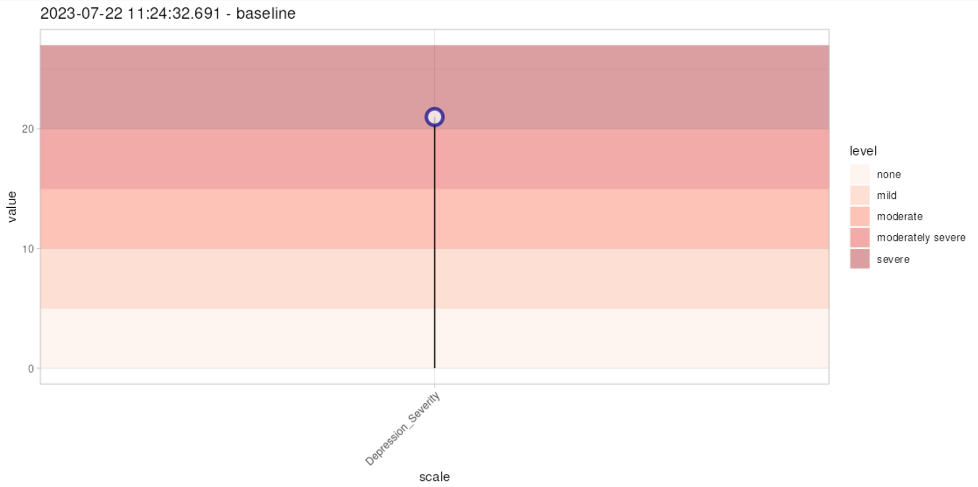

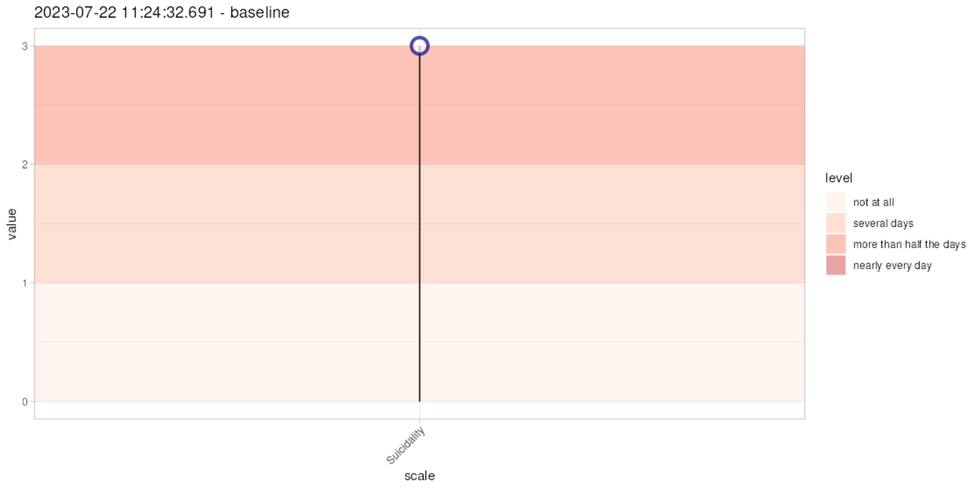

Evaluation

Show 100 **entries** **Search:**

	time	assessment	interpretation	recommendation	warning
1	2023-07-22 11:24:32.691	baseline	The level of depression was severe. The patient reports to be suicidal nearly every day.	Discuss the case with a psychiatrist at your department. Medication might be helpful. Please further evaluate the risk of suicidality and make sure the patient is safe. Consider hospitalisation to keep the patient safe.	⚠

Showing 1 to 1 of 1 entries

Previous 1 Next

Scales

Show 100 **entries** **Search:**

	time	assessment	scale	score	level	cutoffs
1	2023-07-22 11:24:32.691	baseline	Depression_Severity	21	severe	[none: ≥0 & <5]<br>[mild: ≥5 & <10]<br>[moderate: ≥10 & <15]<br>[moderately severe: ≥15 & <20]<br>[severe: ≥20 & <27]
2			Suicidality	3	nearly every day	[not at all: ≥0 & <1]<br>[several days: ≥1 & <2]<br>[more than half the days: ≥2 & <3]<br>[nearly every day: ≥3 & <4]

Showing 1 to 2 of 2 entries

Previous 1 Next

Questionnaire items

Show 100 entries Search:

	Assessment	Answer_time	Question	Selected_choice	Value	Context	ID
	All	All	All	All	All	All	All
1	baseline	2023-07-22T11:24:20Z	Little interest or pleasure in doing things?	More than half the days	2	Over the last two weeks, how often have you been bothered by any of the following problems?	1
2	baseline	2023-07-22T11:24:22Z	Feeling down, depressed, or hopeless?	Several days	1	Over the last two weeks, how often have you been bothered by any of the following problems?	1
3	baseline	2023-07-22T11:24:23Z	Trouble falling or staying asleep, or sleeping too much?	More than half the days	2	Over the last two weeks, how often have you been bothered by any of the following problems?	1
4	baseline	2023-07-22T11:24:24Z	Feeling tired or having little energy?	Nearly every day	3	Over the last two weeks, how often have you been bothered by any of the following problems?	1
5	baseline	2023-07-22T11:24:25Z	Poor appetite or overeating?	More than half the days	2	Over the last two weeks, how often have you been bothered by any of the following problems?	1
6	baseline	2023-07-22T11:24:26Z	Feeling bad about yourself - or that you are a failure or have let yourself or your family down?	More than half the days	2	Over the last two weeks, how often have you been bothered by any of the following problems?	1
7	baseline	2023-07-22T11:24:28Z	Trouble concentrating on things, such as reading the newspaper or watching television?	Nearly every day	3	Over the last two weeks, how often have you been bothered by any of the following problems?	1
8	baseline	2023-07-22T11:24:29Z	Moving or speaking so slowly that other people could have noticed? Or the opposite - being so fidgety or restless that you have been moving around a lot more than usual?	Nearly every day	3	Over the last two weeks, how often have you been bothered by any of the following problems?	1
9	baseline	2023-07-22T11:24:32Z	Thoughts that you would be better off dead, or of hurting yourself in some way?	Nearly every day	3	Over the last two weeks, how often have you been bothered by any of the following problems?	1

This report would normally be displayed in a browser tab and it would be interactive.
