# Supplementary material for: Mental Health Information Reporting Assistant (MHIRA)—an open-source software facilitating evidence-based assessment for clinical services
Source: BMC Psychiatry. 2023 Oct 2;23:706. doi: 10.1186/s12888-023-05201-0 (PMC10544613; doi:10.1186/s12888-023-05201-0)
Supplement: Supplementary file 3 — Additional file 3: Supplementary Information 3. Example of MHIRA report with follow-up. [file 12888_2023_5201_MOESM3_ESM.pdf]

Supplementary Information 3 – Example of MHIRA report with follow-up

MHIRA

medicalRecordNo	initials	age	gender
ML006	ML	25.5	female

PHQ-9

PHQ-9

version: 64bbbbb3a1f9b1002a5a0b67

The PHQ-9 measures depression severity. It is responsive to changes of condition severity over time.

The manual can be found here.

The PHQ-9 was developed by Drs. Robert L. Spitzer, Janet B.W. Williams, Kurt Kroenke and colleagues, with an educational grant from Pfizer Inc. No permission required to reproduce, translate, display or distribute.

Figure

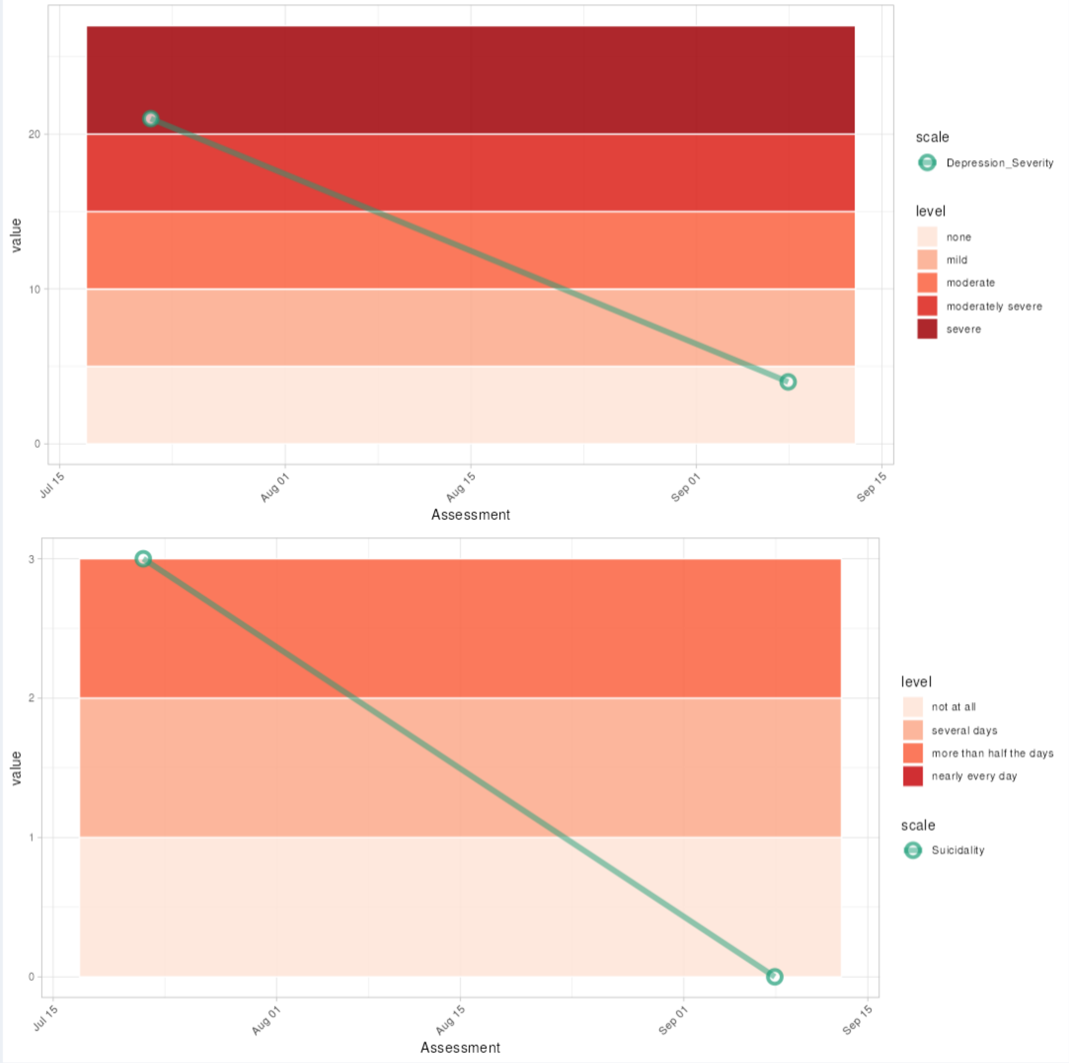

Evaluation

Show 100 entries Search:

	time	assessment	interpretation	recommendation	warning
1	2023-09-08 09:09:11.52	Follow-up	The level of depression was none. The patient does not report to be suicidal according to the PHQ-9.		
2	2023-07-22 11:24:32.691	baseline	The level of depression was severe. The patient reports to be suicidal nearly every day.	Discuss the case with a psychiatrist at your department. Medication might be helpful. Please further evaluate the risk of suicidality and make sure the patient is safe. Consider hospitalisation to keep the patient safe.	⚠

Showing 1 to 2 of 2 entries Previous 1 Next

Scales

Show 100 entries Search:

	time	assessment	scale	score	level	cutoffs
1	2023-09-08 09:09:11.52	Follow-up	Depression_Severity	4	none	[none: ≥0 & <5]<br>[mild: ≥5 & <10]<br>[moderate: ≥10 & <15]<br>[moderately severe: ≥15 & <20]<br>[severe: ≥20 & <27]
2			Suicidality	0	not at all	[not at all: ≥0 & <1]<br>[several days: ≥1 & <2]<br>[more than half the days: ≥2 & <3]<br>[nearly every day: ≥3 & <4]
3	2023-07-22 11:24:32.691	baseline	Depression_Severity	21	severe	[none: ≥0 & <5]<br>[mild: ≥5 & <10]<br>[moderate: ≥10 & <15]<br>[moderately severe: ≥15 & <20]<br>[severe: ≥20 & <27]
4			Suicidality	3	nearly every day	[not at all: ≥0 & <1]<br>[several days: ≥1 & <2]<br>[more than half the days: ≥2 & <3]<br>[nearly every day: ≥3 & <4]

Showing 1 to 4 of 4 entries Previous 1 Next

This report is designed to be viewed in an interactive browser tab. Individual items have been excluded from the table for readability, as including them would result in the table being too large to fit on a single page.
